# Supplementary material for: ProteinShader: illustrative rendering of macromolecules
Source: BMC Struct Biol. 2009 Mar 30;9:19. doi: 10.1186/1472-6807-9-19 (PMC2672931; doi:10.1186/1472-6807-9-19)
Supplement: Additional file 1 — ProteinShader program without source code. This compressed file contains the complete ProteinShader program including associated libraries, but no source code. A README.txt file gives an overview of the ProteinShader distribution, and the index.html file in the help subdirectory has directions on getting started with the program as well as a set of tutorials. [file 1472-6807-9-19-S1.zip › ProteinShader-beta-0_9_4-binary/help/api/org/proteinshader/gui/components/controlpanel/SelectorPanel.html]

SelectorPanel (ProteinShader API)


|  |  |  |
| --- | --- | --- |
| **Overview** \| **Package** \| **Class** \| **Use** \| **Tree** \| **Deprecated** \| **Index** \| **Help** | |  |
| **PREV CLASS**   **NEXT CLASS** | **FRAMES**    **NO FRAMES**     **All Classes** |  |
| SUMMARY: NESTED \| FIELD \| CONSTR \| METHOD | DETAIL: FIELD \| CONSTR \| METHOD |  |


---


## org.proteinshader.gui.components.controlpanel Class SelectorPanel

```
java.lang.Object
  java.awt.Component
      java.awt.Container
          javax.swing.JComponent
              javax.swing.JPanel
                  org.proteinshader.gui.components.controlpanel.SelectorPanel
```

**All Implemented Interfaces:**: ImageObserver, MenuContainer, Serializable, Accessible

---

``` public class SelectorPanel extends JPanel ```

Provides a panel with menus and lists for selecting a Structure's
Models, Chains, Residues, Atoms, Helices, and BetaStrands.

**See Also:**: Serialized Form

---

**Nested Class Summary**

**Nested classes/interfaces inherited from class javax.swing.JPanel**

`JPanel.AccessibleJPanel`

**Nested classes/interfaces inherited from class javax.swing.JComponent**

`JComponent.AccessibleJComponent`

**Nested classes/interfaces inherited from class java.awt.Container**

`Container.AccessibleAWTContainer`

**Nested classes/interfaces inherited from class java.awt.Component**

`Component.AccessibleAWTComponent, Component.BltBufferStrategy, Component.FlipBufferStrategy`


| **Field Summary** | |
| --- | --- |
| `static int` | `DIALOG_HEIGHT`             The height of the Atoms list dialog box. |
| `static int` | `DIALOG_WIDTH`             The width of the Atoms list dialog box. |
| `static Font` | `LIST_FONT`             The font for lists is 14 point plain Courier. |

**Fields inherited from class javax.swing.JComponent**

`accessibleContext, listenerList, TOOL_TIP_TEXT_KEY, ui, UNDEFINED_CONDITION, WHEN_ANCESTOR_OF_FOCUSED_COMPONENT, WHEN_FOCUSED, WHEN_IN_FOCUSED_WINDOW`

**Fields inherited from class java.awt.Component**

`BOTTOM_ALIGNMENT, CENTER_ALIGNMENT, LEFT_ALIGNMENT, RIGHT_ALIGNMENT, TOP_ALIGNMENT`

**Fields inherited from interface java.awt.image.ImageObserver**

`ABORT, ALLBITS, ERROR, FRAMEBITS, HEIGHT, PROPERTIES, SOMEBITS, WIDTH`


**Constructor Summary**

`SelectorPanel(Mediator mediator,
Frame dialogOwner)`
  
          Constructs a SelectorPanel.


| **Method Summary** | |
| --- | --- |
| `int` | `addAtomsToAtomList(Residue residue)`             Adds all Atoms in the Residue to the Atom list. |
| `void` | `clearAtoms()`             Clears the Atom list. |
| `void` | `clearChains()`             Clears the Chain menu, Residue list, and Atom list. |
| `void` | `clearModels()`             Clears all menus and lists. |
| `void` | `clearResidues()`             Clears the Residue list and Atom list. |
| `void` | `closeAtomListDialog()`             Sets the Atom list dialog to invisible and enables the "Atoms" button. |
| `Atom` | `getSelectedAtom()`             Returns the currently selected Atom from the Atom list. |
| `Atom[]` | `getSelectedAtoms()`             Returns an array of the currently selected Atoms from the Atom list. |
| `Chain` | `getSelectedChain()`             Returns the currently selected Chain from the Chain menu. |
| `Model` | `getSelectedModel()`             Returns the currently selected Model from the Model menu. |
| `Residue` | `getSelectedResidue()`             Returns the currently selected Residue from the Residue list. |
| `Residue[]` | `getSelectedResidues()`             Returns an array of the currently selected Residues from the Residue list. |
| `ResidueEnum` | `getSelectedResidueType()`             Returns the currently selected Residue type from the Residue menu. |
| `void` | `openAtomListDialog()`             Sets the Atom list dialog to visible and disables the "Atoms" button. |
| `void` | `setAtomList(Residue residue)`             Sets the Atom list for a Residue. |
| `void` | `setChainMenu(Model model)`             Adds the Model's Chains to the Chain menu. |
| `void` | `setModelMenu(Structure structure)`             Adds the Structure's Models to the Model menu. |
| `void` | `setResidueList(Chain chain)`             Checks the currently selected Residue type (AminoAcids, Heterogens, or Waters) and copies them from the Chain to the Residue list. |

**Methods inherited from class javax.swing.JPanel**

`getAccessibleContext, getUI, getUIClassID, paramString, setUI, updateUI`

**Methods inherited from class javax.swing.JComponent**

`addAncestorListener, addNotify, addVetoableChangeListener, computeVisibleRect, contains, createToolTip, disable, enable, firePropertyChange, firePropertyChange, firePropertyChange, fireVetoableChange, getActionForKeyStroke, getActionMap, getAlignmentX, getAlignmentY, getAncestorListeners, getAutoscrolls, getBorder, getBounds, getClientProperty, getComponentGraphics, getComponentPopupMenu, getConditionForKeyStroke, getDebugGraphicsOptions, getDefaultLocale, getFontMetrics, getGraphics, getHeight, getInheritsPopupMenu, getInputMap, getInputMap, getInputVerifier, getInsets, getInsets, getListeners, getLocation, getMaximumSize, getMinimumSize, getNextFocusableComponent, getPopupLocation, getPreferredSize, getRegisteredKeyStrokes, getRootPane, getSize, getToolTipLocation, getToolTipText, getToolTipText, getTopLevelAncestor, getTransferHandler, getVerifyInputWhenFocusTarget, getVetoableChangeListeners, getVisibleRect, getWidth, getX, getY, grabFocus, isDoubleBuffered, isLightweightComponent, isManagingFocus, isOpaque, isOptimizedDrawingEnabled, isPaintingTile, isRequestFocusEnabled, isValidateRoot, paint, paintBorder, paintChildren, paintComponent, paintImmediately, paintImmediately, print, printAll, printBorder, printChildren, printComponent, processComponentKeyEvent, processKeyBinding, processKeyEvent, processMouseEvent, processMouseMotionEvent, putClientProperty, registerKeyboardAction, registerKeyboardAction, removeAncestorListener, removeNotify, removeVetoableChangeListener, repaint, repaint, requestDefaultFocus, requestFocus, requestFocus, requestFocusInWindow, requestFocusInWindow, resetKeyboardActions, reshape, revalidate, scrollRectToVisible, setActionMap, setAlignmentX, setAlignmentY, setAutoscrolls, setBackground, setBorder, setComponentPopupMenu, setDebugGraphicsOptions, setDefaultLocale, setDoubleBuffered, setEnabled, setFocusTraversalKeys, setFont, setForeground, setInheritsPopupMenu, setInputMap, setInputVerifier, setMaximumSize, setMinimumSize, setNextFocusableComponent, setOpaque, setPreferredSize, setRequestFocusEnabled, setToolTipText, setTransferHandler, setUI, setVerifyInputWhenFocusTarget, setVisible, unregisterKeyboardAction, update`

**Methods inherited from class java.awt.Container**

`add, add, add, add, add, addContainerListener, addImpl, addPropertyChangeListener, addPropertyChangeListener, applyComponentOrientation, areFocusTraversalKeysSet, countComponents, deliverEvent, doLayout, findComponentAt, findComponentAt, getComponent, getComponentAt, getComponentAt, getComponentCount, getComponents, getComponentZOrder, getContainerListeners, getFocusTraversalKeys, getFocusTraversalPolicy, getLayout, getMousePosition, insets, invalidate, isAncestorOf, isFocusCycleRoot, isFocusCycleRoot, isFocusTraversalPolicyProvider, isFocusTraversalPolicySet, layout, list, list, locate, minimumSize, paintComponents, preferredSize, printComponents, processContainerEvent, processEvent, remove, remove, removeAll, removeContainerListener, setComponentZOrder, setFocusCycleRoot, setFocusTraversalPolicy, setFocusTraversalPolicyProvider, setLayout, transferFocusBackward, transferFocusDownCycle, validate, validateTree`

**Methods inherited from class java.awt.Component**

`action, add, addComponentListener, addFocusListener, addHierarchyBoundsListener, addHierarchyListener, addInputMethodListener, addKeyListener, addMouseListener, addMouseMotionListener, addMouseWheelListener, bounds, checkImage, checkImage, coalesceEvents, contains, createImage, createImage, createVolatileImage, createVolatileImage, disableEvents, dispatchEvent, enable, enableEvents, enableInputMethods, firePropertyChange, firePropertyChange, firePropertyChange, firePropertyChange, firePropertyChange, firePropertyChange, getBackground, getBounds, getColorModel, getComponentListeners, getComponentOrientation, getCursor, getDropTarget, getFocusCycleRootAncestor, getFocusListeners, getFocusTraversalKeysEnabled, getFont, getForeground, getGraphicsConfiguration, getHierarchyBoundsListeners, getHierarchyListeners, getIgnoreRepaint, getInputContext, getInputMethodListeners, getInputMethodRequests, getKeyListeners, getLocale, getLocation, getLocationOnScreen, getMouseListeners, getMouseMotionListeners, getMousePosition, getMouseWheelListeners, getName, getParent, getPeer, getPropertyChangeListeners, getPropertyChangeListeners, getSize, getToolkit, getTreeLock, gotFocus, handleEvent, hasFocus, hide, imageUpdate, inside, isBackgroundSet, isCursorSet, isDisplayable, isEnabled, isFocusable, isFocusOwner, isFocusTraversable, isFontSet, isForegroundSet, isLightweight, isMaximumSizeSet, isMinimumSizeSet, isPreferredSizeSet, isShowing, isValid, isVisible, keyDown, keyUp, list, list, list, location, lostFocus, mouseDown, mouseDrag, mouseEnter, mouseExit, mouseMove, mouseUp, move, nextFocus, paintAll, postEvent, prepareImage, prepareImage, processComponentEvent, processFocusEvent, processHierarchyBoundsEvent, processHierarchyEvent, processInputMethodEvent, processMouseWheelEvent, remove, removeComponentListener, removeFocusListener, removeHierarchyBoundsListener, removeHierarchyListener, removeInputMethodListener, removeKeyListener, removeMouseListener, removeMouseMotionListener, removeMouseWheelListener, removePropertyChangeListener, removePropertyChangeListener, repaint, repaint, repaint, resize, resize, setBounds, setBounds, setComponentOrientation, setCursor, setDropTarget, setFocusable, setFocusTraversalKeysEnabled, setIgnoreRepaint, setLocale, setLocation, setLocation, setName, setSize, setSize, show, show, size, toString, transferFocus, transferFocusUpCycle`

**Methods inherited from class java.lang.Object**

`clone, equals, finalize, getClass, hashCode, notify, notifyAll, wait, wait, wait`

**Field Detail**

### LIST\_FONT

```
public static final Font LIST_FONT
```

:   The font for lists is 14 point plain Courier.

---


### DIALOG\_WIDTH

```
public static final int DIALOG_WIDTH
```

:   The width of the Atoms list dialog box.

    **See Also:**: Constant Field Values

---


### DIALOG\_HEIGHT

```
public static final int DIALOG_HEIGHT
```

:   The height of the Atoms list dialog box.

    **See Also:**: Constant Field Values


**Constructor Detail**

### SelectorPanel

```
public SelectorPanel(Mediator mediator,
                     Frame dialogOwner)
```

:   Constructs a SelectorPanel.

    **Parameters:**: `mediator` - the centralized Mediator that most listeners need to call on to accomplish their task.: `dialogOwner` - the owner of any Dialogs opened from the SelectorPanel (or null if there is no requested owner).


**Method Detail**

### getSelectedModel

```
public Model getSelectedModel()
```

:   Returns the currently selected Model from the Model menu.

    :   **Returns:**: The currently selected Model.

---


### getSelectedChain

```
public Chain getSelectedChain()
```

:   Returns the currently selected Chain from the Chain menu.

    :   **Returns:**: The currently selected Chain.

---


### getSelectedResidueType

```
public ResidueEnum getSelectedResidueType()
```

:   Returns the currently selected Residue type from the Residue menu.

    :   **Returns:**: The currently selected Residue type as an ResidueEnum.

---


### getSelectedResidue

```
public Residue getSelectedResidue()
```

:   Returns the currently selected Residue from the Residue list. If
    there are multiple Residues selected, this method returns only the
    first. If there are no Residues selected, null is returned.

    :   **Returns:**: The currently selected Residue.

---


### getSelectedResidues

```
public Residue[] getSelectedResidues()
```

:   Returns an array of the currently selected Residues from the
    Residue list. If no Residues are selected, the array will have a
    length of zero.

    :   **Returns:**: The currently selected Residues as an array.

---


### getSelectedAtom

```
public Atom getSelectedAtom()
```

:   Returns the currently selected Atom from the Atom list. If there
    are multiple Atoms selected, this method returns only the first.
    If there are no Atoms selected, null is returned.

    :   **Returns:**: The currently selected Atom.

---


### getSelectedAtoms

```
public Atom[] getSelectedAtoms()
```

:   Returns an array of the currently selected Atoms from the Atom
    list. If no Atoms are selected, the array will have a length of
    zero.

    :   **Returns:**: The currently selected Atoms as an array.

---


### setModelMenu

```
public void setModelMenu(Structure structure)
```

:   Adds the Structure's Models to the Model menu. The first Model
    will be set selected. If Structure is null or has no Models, then
    the Model menu will be blank and the number of Models will be
    shown as 0.

    :   **Parameters:**: `structure` - the Structure holding the Models to add to the menu.

---


### setChainMenu

```
public void setChainMenu(Model model)
```

:   Adds the Model's Chains to the Chain menu. The first Chain will
    be set selected. If Model is null or has no Chains, then the
    Chain menu will be blank and the number of Chains will be shown
    as 0.

    :   **Parameters:**: `model` - the Model holding the Chains to add to the menu.

---


### setResidueList

```
public void setResidueList(Chain chain)
```

:   Checks the currently selected Residue type (AminoAcids,
    Heterogens, or Waters) and copies them from the Chain to the
    Residue list. The list will be empty if the Chain does not have
    any of the requested type of Residue.

    :   **Parameters:**: `chain` - the Chain holding the Residues to add to the Residue list.

---


### setAtomList

```
public void setAtomList(Residue residue)
```

:   Sets the Atom list for a Residue.

    :   **Parameters:**: `residue` - the Chain holding the Residues to add to the Residue list.

---


### openAtomListDialog

```
public void openAtomListDialog()
```

:   Sets the Atom list dialog to visible and disables the "Atoms"
    button.

---


### closeAtomListDialog

```
public void closeAtomListDialog()
```

:   Sets the Atom list dialog to invisible and enables the "Atoms"
    button.

---


### clearModels

```
public void clearModels()
```

:   Clears all menus and lists. Any labels reporting the number of
    items in a menu or list is set to zero.

---


### clearChains

```
public void clearChains()
```

:   Clears the Chain menu, Residue list, and Atom list.

---


### clearResidues

```
public void clearResidues()
```

:   Clears the Residue list and Atom list.

---


### clearAtoms

```
public void clearAtoms()
```

:   Clears the Atom list.

---


### addAtomsToAtomList

```
public int addAtomsToAtomList(Residue residue)
```

:   Adds all Atoms in the Residue to the Atom list. The label with
    the number of Atoms will be updated, and the total number of items
    now in the list will be returned.

    :   **Parameters:**: `residue` - the Residue holding the Atoms. **Returns:**: The total number of items in the Atom list.


---


|  |  |  |
| --- | --- | --- |
| **Overview** \| **Package** \| **Class** \| **Use** \| **Tree** \| **Deprecated** \| **Index** \| **Help** | |  |
| **PREV CLASS**   **NEXT CLASS** | **FRAMES**    **NO FRAMES**     **All Classes** |  |
| SUMMARY: NESTED \| FIELD \| CONSTR \| METHOD | DETAIL: FIELD \| CONSTR \| METHOD |  |


---

# *Copyright © 2007-2008*
